# 

**Published:** 1880-04

**Authors:** 


### EDITORIAL.

### WANTED.

The following numbers of the Dental Register are wanted to complete volumes for binding, viz:

February and September Nos. of vol. 18, year 1864. February, May, June, July and September Nos. of vol. 19, year 1864. April, May, June, July, November and December Nos. of 1877.

I will be glad to receive any or all of these numbers, for which 25 cents each will be paid.

Address,

J. TAFT, Cincinnati, O.

### TO THE AMERICAN DENTAL ASSOCIATION.

An effort is being made and with strong probability of success, to arrange for an excursion to the American Dental Association, at Boston, about the 1st of August next, by a very attractive and interesting route, on which will be many of the most interesting points in the country, and at a very low rate, perhaps not over $25 for the round trip. The arrangements are not yet completed, but will probably be given in the May Register.

### TENNESSEE DENTAL SOCIETY.

The annual meeting of the Tennessee State Dental Society, will be held in the city of Nashville, on the 17th day of May, 1880.

A very large meeting is expected and desired. It is important that the entire profession in the State, so far as possible, be present: and a cordial invitation is extended to the profession outside of the State to be present. There are professional interests now at Nashville that should draw together a large number of the profession.

### NORTHERN OHIO DENTAL ASSOCIATION.

The twenty-first annual meeting will be held in the city of Akron, Ohio, Tuesday and Wednesday, May 11th and 12, 1880, commencing at 10 A. M., Tuesday.

SUBJECTS FOR DISCUSSION.

1. Present Status of Filling Teeth.
2. Periodontites: Cause, Prognosis and Treatment.
3. Tumors of the Mouth.
4. Cases in Practice.

During the session, Clinics by Drs. Palmer and Butler.

A cordial invitation is extended to all the profession.

### ILLINOIS STATE DENTAL SOCIETY.

THE sixteenth annual meeting of the Illinois State Dental Society will be held at Bloomington, on Tuesday, May 11th, and continue four days. Dentists from other states are cordially invited to be present.

EDMUND NOYES, Secretary.

### CONTENTS.

### COMMUNICATIONS.

### THE DENTAL REGISTER,

A MONTHLY MAGAZINE DEVOTED TO THE INTERESTS OF

**THE DENTAL PROFESSION.**

*TERMS REDUCED TO $2.50 PER YEAR. SINGLE COPIES 25C.*

EDITED BY PROF. J. TAFT, D.D.S.

AND

*Published by SPENCER, CROCKER & CO., Ohio Dental Depot.*

The volume commences in January and closes in December.

The REGISTER being issued monthly is always fresh, therefore Indispensable to the practitioner who desires to keep posted up with the new inventions and improvements which are daily developed. Neither expense nor effort will be spared to give value and variety to its contents. Articles will be illustrated with well executed engravings whenever they will add to the interest or assist to explanation.

Its extensive circulation and frequency of issue make it one of the BEST DENTAL ADVERTISING MEDIUMS in the United States.

All subscriptions must begin with January or July.

Local or special notices, five lines or less, will be inserted for$3 each insertion; over five lines, 50 cts. per line.

All advertising bills payable in advance, or at furthest, after the issue of the first number containing the advertisement. All trânsient advertisements to be paid for in advance.

☞CONTRIBUTIONS SOLICITED.

Exclusively Editorial Communications should be addressed to Editor.

The latest number of the REGISTER may be ordered with or without oth-goods at any time, and all letters should be addressed to

*SPENCER, CROCKER & CO., Ohio Dental Depot, Cincinnati, O.*

